# Circadian clocks, rhythmic synaptic plasticity and the sleep-wake cycle in zebrafish

**DOI:** 10.3389/fncir.2013.00009

**Published:** 2013-02-01

**Authors:** Idan Elbaz, Nicholas S. Foulkes, Yoav Gothilf, Lior Appelbaum

**Affiliations:** ^1^The Mina and Everard Goodman Faculty of Life Sciences, The Leslie and Susan Gonda Multidisciplinary Brain Research Center, Bar-Ilan UniversityRamat-Gan, Israel; ^2^Karlsruhe Institute of Technology, Campus North, Institute of Toxicology and GeneticsEggenstein-Leopoldshafen, Germany; ^3^Department of Neurobiology, George S. Wise Faculty of Life Sciences, Sagol School of Neurosciences, Tel Aviv UniversityTel Aviv, Israel

**Keywords:** zebrafish, circadian rhythms, synaptic plasticity, circadian clock, sleep, hypocretin, orexin, melatonin

## Abstract

The circadian clock and homeostatic processes are fundamental mechanisms that regulate sleep. Surprisingly, despite decades of research, we still do not know why we sleep. Intriguing hypotheses suggest that sleep regulates synaptic plasticity and consequently has a beneficial role in learning and memory. However, direct evidence is still limited and the molecular regulatory mechanisms remain unclear. The zebrafish provides a powerful vertebrate model system that enables simple genetic manipulation, imaging of neuronal circuits and synapses in living animals, and the monitoring of behavioral performance during day and night. Thus, the zebrafish has become an attractive model to study circadian and homeostatic processes that regulate sleep. Zebrafish clock- and sleep-related genes have been cloned, neuronal circuits that exhibit circadian rhythms of activity and synaptic plasticity have been studied, and rhythmic behavioral outputs have been characterized. Integration of this data could lead to a better understanding of sleep regulation. Here, we review the progress of circadian clock and sleep studies in zebrafish with special emphasis on the genetic and neuroendocrine mechanisms that regulate rhythms of melatonin secretion, structural synaptic plasticity, locomotor activity and sleep.

## Introduction

All organisms demonstrate a wide variety of physiological, biochemical and behavioral daily rhythms that are driven by a highly conserved endogenous timing mechanism, the circadian clock. The maintenance and synchronization of this clock and the concurrent rhythms constitute an adaptive advantage, and its disruption in humans has been associated with physiological and mental disorders. A well-studied output of the circadian clock is the sleep-wake cycle. Sleep is a highly conserved process (Hartse, 2011) although its function remains one of the biggest mysteries in science (Cirelli and Tononi, 2008; Mignot, 2008). Theories that attempt to explain the role of sleep range from ecological considerations and energy conservation to synaptic plasticity and memory consolidation (Saper et al., 2005; Siegel, 2005; Nishino and Sakurai, 2006; Tononi and Cirelli, 2006; Cirelli, 2009; Sehgal and Mignot, 2011; Wang et al., 2011). The sleep state is associated with cycles of electroencephalograph (EEG) patterns (primarily in mammals), a species-specific sleep posture, a period of reversible quiescence, and decreased levels of sensory awareness to external stimuli. Sleep is regulated both by the circadian clock, which sets the timing of sleep, and by homeostatic mechanisms, as indicated by a compensatory increase in the intensity and duration of sleep after sleep deprivation (SD).

In mammals, including humans, sleep and other circadian rhythms are driven by a master oscillator that resides in the suprachiasmatic nucleus (SCN) of the hypothalamus (Reppert et al., 1981; Granados-Fuentes and Herzog, 2012). Among the many targets that are controlled by the mammalian SCN are hormonal and neuronal circuits that, in turn, feedback on the master oscillator and influence sleep/wake cycles. These include the rhythmic production of melatonin in the pineal gland and rhythmic secretion of neuropeptides and monoamines in the brain (Morris et al., 2012). Melatonin is secreted only during the night in all vertebrates. It affects the activity of the SCN, where the expression of melatonin receptors is enriched, and in diurnal birds and fish, it is a strong sleep-promoting hormone (Zhdanova, 2005). Another sleep/wake regulatory factor is the hypothalamic neuropeptide hypocretin/orexin (HCRT). Loss of HCRT neurons is associated with the sleep disorder narcolepsy, which is characterized by excessive daytime sleepiness, fragmentation of sleep during the night and cataplexy (brief loss of muscle tone triggered by emotional stimuli) (Lin et al., 1999; Nishino and Sakurai, 2006; Adamantidis and De Lecea, 2008).

The zebrafish offers many advantages for studying the circadian clock and the regulation of sleep. It is amenable to high throughput genetic and behavioral experiments, and its early developmental stages are transparent, enabling neuronal imaging *in vivo*. The complex neuro-regulatory mechanisms and sleep regulating nuclei underlying sleep/wake cycles in mammals are conserved, but much simpler in zebrafish. For example, the zebrafish HCRT neuronal circuits are similar in function and anatomy to mammals (Panula, 2010), but are represented by small number of neurons in the zebrafish brain (Faraco et al., 2006). The pineal gland in zebrafish develops remarkably early (Vatine et al., 2011), is photoreceptive and contains an intrinsic circadian oscillator that directs melatonin rhythms. Thus, the pineal gland is considered a central circadian pacemaker that conveys circadian timing information to physiological and behavioral processes. In this review, we describe the progress of circadian and sleep studies in zebrafish with special emphasis on their neuroendocrine regulation.

## The circadian clock system in zebrafish

One of the most studied outputs of the circadian clock in vertebrates is the melatonin rhythm. The zebrafish pineal gland drives rhythms of melatonin-independent of any neuronal input or other master clock structures (Cahill, 1996; Noche et al., 2011). The aralkylamine-*N*-acetyltransferase (*aanat*) gene encodes the key enzyme of melatonin synthesis. Zebrafish *aanat2* expression and melatonin synthesis begin remarkably early, within 1 day post fertilization (dpf), and exhibit circadian clock-controlled rhythms at 2 dpf (Gothilf et al., 1999; Kazimi and Cahill, 1999). Genetic investigations of the pineal circadian clock mechanisms and its functional development have revealed that light and light-induced genes are required for the onset of the core molecular oscillator in the pineal gland (Ziv et al., 2005; Vuilleumier et al., 2006; Vatine et al., 2011). Extensive studies performed by Zhdanova and co-workers on the role of melatonin in zebrafish indicate that melatonin is a sleep-promoting agent (Zhdanova, 2011). Melatonin was also shown to affect memory acquisition (Rawashdeh et al., 2007), and to schedule the timing of reproduction (Carnevali et al., 2011) and feeding (Piccinetti et al., 2010).

Another important feature of the zebrafish circadian clock system is that light-entrainable circadian oscillators exist in all organs and even in cell cultures (Whitmore et al., 2000; Pando et al., 2001). Zebrafish cell lines have been used to study the role of the different clock genes within the core oscillator (Vallone et al., 2004, 2005) revealing that similar mechanisms constitute the core molecular oscillator in central and peripheral clocks. Current and future studies combining functional analysis of clock genes in living animals and in the light-entrainable, clock-containing zebrafish cell lines will enhance our understanding of the molecular mechanisms underlying the circadian clock and its entrainment (Tamai et al., 2005, 2007; Carr et al., 2006; Vatine et al., 2009).

Monitoring rhythms of locomotor activity is very frequently used to measure circadian clock output. Being a diurnal species, adult zebrafish demonstrate locomotor activity that peaks during the day (Hurd et al., 1998). The larvae start to exhibit a stable diurnal rhythm of locomotor activity at 4 dpf (Hurd and Cahill, 2002). An important hallmark of a circadian clock-driven rhythm is that it persists under constant photic conditions. Indeed, adult zebrafish also exhibit rhythmic activity and increase activity during the subjective day under constant dark (DD) conditions (Cahill et al., 1998; Hurd et al., 1998). Similarly, zebrafish larvae are rhythmic under DD (Hurd and Cahill, 2002) or constant dim light (Appelbaum et al., 2009, 2010; Tovin et al., 2012). It should be noted, however, that zebrafish are directly influenced by the photic conditions, which promote constant activity; i.e., “masking effects.” Thus, while robust rhythms of locomotor activity are detected under light/dark cycles (LD), under constant light (LL) most individuals are constantly active, which also leads to a complete loss of their rhythms. Likewise, under DD, locomotor activity is reduced to the point in which rhythms are lost in some individuals, therefore, constant dim light has been used (Tovin et al., 2012). Rhythmic locomotor activity clearly reflects an integration of environmental effects and regulation by intrinsic central and peripheral circadian clocks. As sleep/wake cycles are a key output of the circadian clock, the extent to which rhythmic locomotor activity reflects sleep and wakefulness was studied in larvae and adults.

## Sleep in zebrafish

### Using behavioral criteria to measure the sleep state in zebrafish

Sleep has been examined in various fish species either in the natural environment or in laboratory conditions. During sleep, fish exhibit place preference, reduced heart and respiratory rates, typical sleep-postures and reduced sensitivity to external stimuli such as food, electric current or mechanical contact (Tauber et al., 1969; Shapiro and Hepburn, 1976; Campbell and Tobler, 1984; Tobler and Borbely, 1985; Goldshmid et al., 2004). The zebrafish has been established as a promising model for sleep and sleep disorder research (Zhdanova et al., 2001; Prober et al., 2006; Yokogawa et al., 2007; Appelbaum et al., 2009; Rihel et al., 2010; Sigurgeirsson et al., 2011; Elbaz et al., 2012). Since its small size and the water habitat preclude EEG measurements, behavioral criteria are used to distinguish sleep and wake states in zebrafish. Notably, in infant mammals, before the differentiation of EEG (when sleep state-dependent neocortical activity is absent), sleep is reliably characterized by the presence of tonic and phasic muscle tone (Karlsson and Blumberg, 2005; Karlsson et al., 2005). Therefore, as determined for other small non-mammalian species (Hendricks et al., 2000; Raizen et al., 2008), the key behavioral criteria for sleep are: (1) a period of immobility that is associated with a specific posture; (2) quick reversibility to wakefulness (distinguishes sleep from coma or hibernation); (3) increased arousal threshold to external stimuli (indication of low level of sensory awareness); (4) sleep-rebound after SD (indication of homeostatic regulation), and (5) preference for nocturnal or diurnal sleep (indication of circadian regulation) (Zimmerman et al., 2008). These behavioral criteria were used to show that a minimum of 1 min of immobility is associated with elevated arousal threshold and a sleep-like state in 5–7 dpf larvae (Prober et al., 2006; Elbaz et al., 2012). In these studies, arousal was stimulated by pulses of light, a procedure which may not be ideal because retinal responsiveness is reduced at night (Emran et al., 2010). However, responsiveness to changes in light intensity is also mediated by extra-retinal photoreceptors (Fernandes et al., 2012). In adults, an electrical stimulus, rather than light, was used to set the arousal threshold and define sleep as a minimum of 6 sec of immobility (Yokogawa et al., 2007). Thus, as in the fly where 5 min of immobility was defined as a sleep-like state (Hendricks et al., 2000), an array of behavioral experiments was used to define sleep in zebrafish. However, since EEG is not applicable in zebrafish, additional techniques should be applied to differentiate rest from sleep. For example, sleep in zebrafish was also studied using *c-fos* expression (Appelbaum et al., 2010; Elbaz et al., 2012), a well-established marker for wakefulness (Cirelli and Tononi, 2000). Recently developed techniques for measuring neuron activity via genetically encoded calcium sensors in the whole brain of live larvae (Ahrens et al., 2012) or by rapid bioluminescent signals in genetically specified neurons of free swimming zebrafish (Naumann et al., 2010) promise to provide a causal link between neural activity and the state of sleep or wakefulness.

### Homeostatic and circadian regulation of sleep in zebrafish

**Figure 1:**
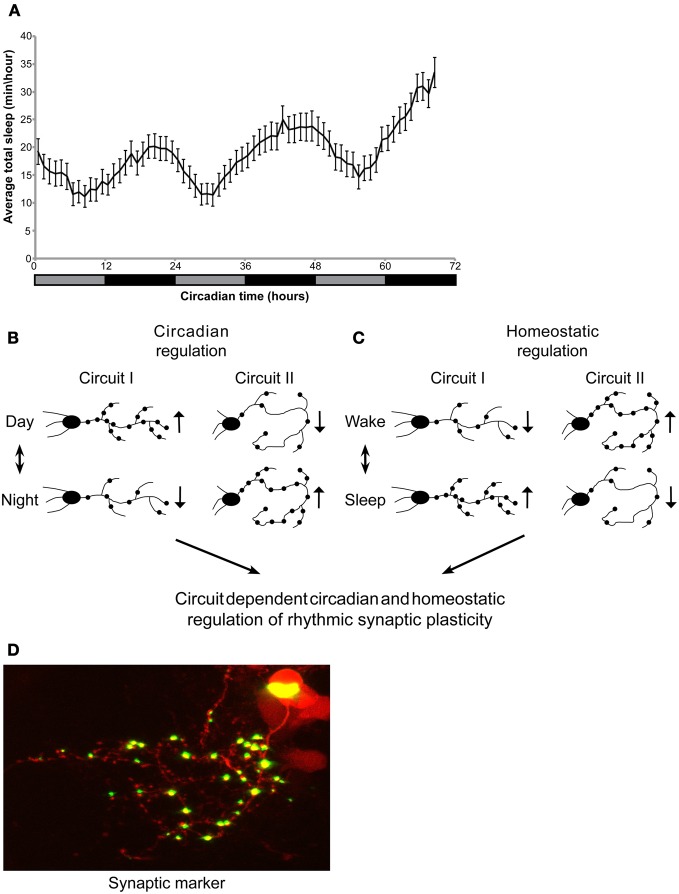
**Circadian regulation of sleep in larvae. Circadian and homeostatic (sleep-dependent) regulation of circuit-dependent rhythmic structural synaptic plasticity. (A)** Zebrafish larvae were kept under LD for 6 days. At 6-8 dpf, sleep was monitored under constant dim light for three consecutive days (gray and black bar represent subjective day and night, respectively). Sleep was defined and monitored as previously described (Elbaz et al., 2012). Sleep time was rhythmic and peaked during the night (*n* = 55). **(B,C)** A proposed model demonstrating circadian and sleep/wake regulation of structural synaptic plasticity in the brain. Rhythmicity of synapse number, size and location is affected by: **(B)** the circadian clock **(C)** homeostatic process (sleep and wake). **(B)** While the number of synapses in a given circuit I increase during the day, the circadian clock could drive, at the same time, a reduction in synapse number in circuit II. **(C)** In parallel, homeostatic process controls the number of synapses in both circuits I and II. These two processes may be opposed or additive. Thus, the identity and role of a specific circuit determines its relative regulation by the circadian and homeostatic processes. **(D)** Imaging of synaptic fluorescence marker in live zebrafish larvae. This technique enables monitoring of structural synaptic plasticity in specific circuit during day and night, sleep and wakefulness.

Adult zebrafish sleep mainly during the night under both LD and DD, indicating circadian clock regulation of the sleep/wake cycle. In contrast, under LL, light seems to suppress sleep, and rhythms of sleep/wake behavior disappear, reflecting the masking effect of light. Indeed, in adults kept under LL, sleep-like behavior could be noted only after 1 week (Yokogawa et al., 2007). Similarly, zebrafish larvae also demonstrate a rhythmic sleep/wake cycle, under LD (Elbaz et al., 2012). However, the sleep/wake cycle was not examined in larvae under constant conditions. Here, we show circadian rhythms of sleep/wake cycles under constant dim light, indicating that sleep is regulated by the circadian clock in 6–8 dpf larvae (Figure 1A).

The timing of sleep is mainly controlled by the circadian clock, however, in all animals sleep is also regulated by a homeostatic mechanism. SD is followed by sleep-rebound that is independent of the circadian time. This has been revealed in studies of adult zebrafish that were sleep deprived by electrical stimulation during the 6 h of the dark prior to usual light onset, and then released into the subjective day. Under the dark, a sleep-rebound was observed, indicating homeostatic regulation of sleep (Yokogawa et al., 2007). Homeostatic control of sleep in zebrafish larvae was first demonstrated by Zhdanova and colleagues. Six hours of SD, induced by constant vibration, increased sleep time during the following subjective day (Zhdanova et al., 2001). More recently, a similar but more moderate protocol was used to uncovered a subtle behavioral phenotype in a zebrafish model for narcolepsy (Elbaz et al., 2012).

### Neural networks that regulate sleep and wakefulness in zebrafish

Several networks regulate sleep and wakefulness in mammals including aminergic, cholinergic, GABAergic and hypocretinergic systems. The organization and role of these networks is conserved in zebrafish (Panula et al., 2010). Furthermore, the zebrafish offers many advantages for high throughput, whole animal pharmacological screens since compounds can be delivered easily by simply dissolving them into the culture water of individual embryos (Rihel et al., 2010). Thus, the zebrafish larva emerges as a promising model to dissect the neuronal networks that regulate sleep using chemical genetics and to search for putative pharmacological sleep regulators.

The HCRT is an example of a neuronal network that has been a subject of intense studies in zebrafish, primarily because of its association with narcolepsy. Only 16–40 HCRT neurons, located in the lateral hypothalamus, innervate wide areas within the zebrafish brain (Kaslin et al., 2004; Faraco et al., 2006; Prober et al., 2006; Yokogawa et al., 2007; Appelbaum et al., 2009). To understand the role of HCRT in zebrafish, several genetic strategies have been developed including inducible global HCRT overexpression (Prober et al., 2006), mutation of the HCRT receptor, HCRTR (Yokogawa et al., 2007), expression of the Ca^2+^-sensitive photoprotein GFP-apoAequorin in HCRT neurons (Naumann et al., 2010), and genetic ablation of HCRT neurons (Elbaz et al., 2012). These studies have showed that HCRT neurons regulate both wake and sleep and are most important during sleep/wake transitions (Table 1). Interestingly, HCRT neuron-ablated larvae increase sleep during the day and demonstrate fragmented sleep during the night, consistent with the results observed under HCRT overexpression and HCRTR mutation, respectively (Table 1). This function may be mediated by a hypothalamic-pineal gland circuit, which regulates HCRT and melatonin secretion (Appelbaum et al., 2009).

**Table 1:** **HCRT neurons control behavioral sleep-wake transitions**.

**Genetic manipulation**	**Developmental stage**	**Circadian time**	**Sleep time**	**Sleep/Wake transitions**	**References**
HCRT mRNA over-expression	Larvae	Day	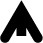	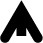	Prober et al., 2006
		Night	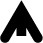	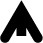		
HCRT receptor mutant	Adult	Day	n.e.	n.e.	Yokogawa et al., 2007
		Night	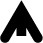	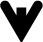		
HCRT neuron-ablation	Larvae	Day	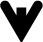	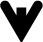	Elbaz et al., 2012
		Night	n.e.	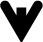		

HCRT over-expression in larvae reduced sleep time and sleep-wake transitions both during the day and night (Prober et al., 2006). In the adult HCRT receptor mutant (HCRTR^-/-^; Yokogawa et al., 2007), sleep time was reduced and fragmented during the night. However, during the day no effect (n.e.) on sleep was observed. In both cases, genetic manipulation of the HCRT system altered sleep/wake transitions, and the apparent contradictory effect on sleep time may reflect larval vs. adult behavior. In agreement with these observations, ablation of HCRT neurons increased sleep during the day, and increase sleep/wake transitions during day and night (Elbaz et al., 2012). Thus, the most profound and consistent behavioral role of HCRT neurons is the regulation of behavioral sleep/wake state transitions. Indeed, HCRT neurons are most active during the transition in locomotor activity (Naumann et al., 2010).

## Circadian and homeostatic sleep-dependent control of structural synaptic plasticity

To synchronize physiology and behavior with the daily cycle, the circadian clock acts at different levels, ranging from the control of rhythmic gene expression, protein degradation and transportation, to the modification of the structure of neuronal circuits and synapses. While circadian control of the expression of genes and proteins has been studied extensively (Bass and Takahashi, 2010), data on the circadian regulation of synaptic plasticity and how this, in turn, controls circuit function and rhythmic behavior is limited (Frenkel and Ceriani, 2011). Species with a simple nervous system provide an ideal platform to study rhythmic structural synaptic plasticity that is associated with behavior (Wang et al., 2011). “Structural” synaptic plasticity is defined here as changes in the size, shape, orientation, and number of inhibitory or excitatory synapses. In *Drosophila*, several studies have demonstrated that the circadian clock controls daily changes in neuronal and synaptic structure (Mehnert et al., 2007; Fernandez et al., 2008; Pyza and Gorska-Andrzejak, 2008; Damulewicz and Pyza, 2011). It is imperative that findings in the fruit fly are extended to assess the regulation and role of rhythmic structural synaptic plasticity in vertebrate models where it is possible to monitor multiple excitatory and inhibitory neuronal circuits in live animals. The genetic and live imaging tools available for the zebrafish make this model particularly attractive for this task. Indeed, using synaptic fluorescence markers (Niell et al., 2004; Meyer and Smith, 2006) and time-lapse two photon imaging, rhythmic synaptic plasticity was monitored in live larvae. Visualizing synapses in transgenic lines that express the pre-synaptic protein, synaptophysin (SYP), fused to EGFP, revealed that the number of synapses along HCRT axons follow a diurnal rhythm under both LD and DD conditions (Appelbaum et al., 2010). This data suggest that the circadian clock regulate structural synaptic plasticity, a hypothesis that can be directly tested in zebrafish mutants for clock genes.

Although the data above indicate circadian control of structural synaptic plasticity, homeostatic sleep-dependent process should also be considered as regulators of rhythmic neuronal plasticity. In flies, brain-wide quantification of proteins that are associated with synaptic potentiation and circuit-specific imaging of synaptic terminals showed that the levels of synaptic components are high during wakefulness and low during sleep (Donlea et al., 2009; Gilestro et al., 2009). In zebrafish, two-photon imaging of fluorescent synaptic markers revealed that rhythms of structural synaptic plasticity in HCRT axons are mainly regulated by the circadian clock. Nevertheless, a minor, yet significant effect of SD on synapse number was also demonstrated, indicating a homeostatic control of synaptic density (Appelbaum et al., 2010). To further understand the effect of sleep on brain plasticity, time-lapse imaging of several circuits under sleep-promoting drugs or in genetically manipulated sleep mutants, such as the HCRT neuron-ablated larvae (that demonstrate fragmented sleep, Elbaz et al., 2012), could provide significant data that link the sleep/wake cycle with circuit modifications. Based on the current limited data, we proposed a model for combined circadian and homeostatic regulation of rhythmic structural synaptic plasticity. The balance between these processes is expected to vary significantly among circuits and may be opposed or additive, depending on the role of the specific circuit. For example, brain regions, such as the hypothalamus, that regulate fundamental behavioral rhythms (such as feeding, sleep, and wake activity) would exhibit mainly clock-controlled synaptic plasticity with minor homeostatic effect (as for the HCRT axons, Appelbaum et al., 2010). In contrast, brain regions that mediate experience-dependent behavior (such as learning and memory) would demonstrate mainly sleep-dependent structural synaptic plasticity (Figures 1B–D). Thus, the brain undergoes significant circuit and synaptic changes during the circadian cycle as well as during sleep and wake episodes.

## Future directions and concluding remarks

Clearly advances in genetic and imaging tools will play a key role in the future application of zebrafish to study sleep and clock regulation within the nervous system. Genetic bipartite methods for refined neuronal gene targeting, such as the UAS/Gal4 system, are routinely used in zebrafish (Scott et al., 2007; Asakawa and Kawakami, 2008; Vatine et al., 2013). Application of this technique to image synapses in many brain circuits will provide a powerful future approach. Real-time imaging of synaptic markers in a specific circuit in the zebrafish brain during day and night and after SD will shed light on how circadian and homeostatic processes regulate synaptic plasticity. A limitation of this approach is that anatomical changes of fluorescence synaptic markers do not necessarily represent synaptic transmission and neuronal activity. Monitoring structural synaptic plasticity in correlation with behavior in the same individual fish can partially overcome this limitation. Moreover, imaging of genetically modified calcium indicators fused to synaptic markers (Dreosti et al., 2009) that can identify locations and activity of synapses, simultaneously, in the living animal, could provide a complete solution.

What is so important about sleep that warrants the risk of being at a reduced state of awareness? To answer this fundamental question, a critical challenge is to visualize circadian- and sleep-related circuits in the living brain, which contains an incomprehensible, dense population of sleep and wake regulatory neurons and their processes. The zebrafish is a vertebrate model, which provides a unique opportunity to look into a relatively simple nervous system, which retains the fundamental sleep- and clock-regulating circuits.

### Conflict of interest statement

The authors declare that the research was conducted in the absence of any commercial or financial relationships that could be construed as a potential conflict of interest.

## References

[B1] Adamantidis A. De Lecea L. (2008). Sleep and metabolism: shared circuits, new connections. Trends Endocrinol. Metab. 19, 362–370 10.1016/j.tem.2008.08.007 18938086

[B2] Ahrens M. B. Li J. M. Orger M. B. Robson D. N. Schier A. F. Engert F. (2012). Brain-wide neuronal dynamics during motor adaptation in zebrafish. Nature 485, 471–477 10.1038/nature11057 22622571 PMC3618960

[B3] Appelbaum L. Wang G. Yokogawa T. Skariah G. M. Smith S. J. Mourrain P. (2010). Circadian and homeostatic regulation of structural synaptic plasticity in hypocretin neurons. Neuron 68, 87–98 10.1016/j.neuron.2010.09.006 20920793 PMC2969179

[B4] Appelbaum L. Wang G. X. Maro G. S. Mori R. Tovin A. Marin W. (2009). Sleep-wake regulation and hypocretin-melatonin interaction in zebrafish. Proc. Natl. Acad. Sci. U.S.A. 106, 21942–21947 10.1073/pnas.906637106 19966231 PMC2799794

[B5] Asakawa K. Kawakami K. (2008). Targeted gene expression by the Gal4-UAS system in zebrafish. Dev. Growth Differ. 50, 391–399 10.1111/j.1440-169X.2008.01044.x 18482403

[B6] Bass J. Takahashi J. S. (2010). Circadian integration of metabolism and energetics. Science 330, 1349–1354 10.1126/science.1195027 21127246 PMC3756146

[B7] Cahill G. M. (1996). Circadian regulation of melatonin production in cultured zebrafish pineal and retina. Brain Res. 708, 177–181 10.1016/0006-8993(95)01365-2 8720875

[B8] Cahill G. M. Hurd M. W. Batchelor M. M. (1998). Circadian rhythmicity in the locomotor activity of larval zebrafish. Neuroreport 9, 3445–3449 9855296 10.1097/00001756-199810260-00020

[B9] Campbell S. S. Tobler I. (1984). Animal sleep: a review of sleep duration across phylogeny. Neurosci. Biobehav. Rev. 8, 269–300 6504414 10.1016/0149-7634(84)90054-x

[B10] Carnevali O. Gioacchini G. Maradonna F. Olivotto I. Migliarini B. (2011). Melatonin induces follicle maturation in Danio rerio. PLoS ONE 6:e19978 10.1371/journal.pone.0019978 21647435 PMC3102064

[B11] Carr A. J. Tamai T. K. Young L. C. Ferrer V. Dekens M. P. Whitmore D. (2006). Light reaches the very heart of the zebrafish clock. Chronobiol. Int. 23, 91–100 10.1080/07420520500464395 16687283

[B12] Cirelli C. (2009). The genetic and molecular regulation of sleep: from fruit flies to humans. Nat. Rev. Neurosci. 10, 549–560 10.1038/nrn2683 19617891 PMC2767184

[B13] Cirelli C. Tononi G. (2000). On the functional significance of c-fos induction during the sleep-waking cycle. Sleep 23, 453–469 10875553

[B14] Cirelli C. Tononi G. (2008). Is sleep essential? PLoS Biol. 6:e216 10.1371/journal.pbio.0060216 18752355 PMC2525690

[B15] Damulewicz M. Pyza E. (2011). The clock input to the first optic neuropil of Drosophila melanogaster expressing neuronal circadian plasticity. PLoS ONE 6:e21258 10.1371/journal.pone.0021258 21760878 PMC3124489

[B16] Donlea J. M. Ramanan N. Shaw P. J. (2009). Use-dependent plasticity in clock neurons regulates sleep need in Drosophila. Science 324, 105–108 10.1126/science.1166657 19342592 PMC2850598

[B17] Dreosti E. Odermatt B. Dorostkar M. M. Lagnado L. (2009). A genetically encoded reporter of synaptic activity *in vivo*. Nat. Methods 6, 883–889 10.1038/nmeth.1399 19898484 PMC2859341

[B18] Elbaz I. Yelin-Bekerman L. Nicenboim J. Vatine G. Appelbaum L. (2012). Genetic ablation of hypocretin neurons alters behavioral state transitions in zebrafish. J. Neurosci. 32, 12961–12972 10.1523/JNEUROSCI.1284-12.2012 22973020 PMC6703801

[B19] Emran F. Rihel J. Adolph A. R. Dowling J. E. (2010). Zebrafish larvae lose vision at night. Proc. Natl. Acad. Sci. U.S.A. 107, 6034–6039 10.1073/pnas.0914718107 20224035 PMC2851871

[B20] Faraco J. H. Appelbaum L. Marin W. Gaus S. E. Mourrain P. Mignot E. (2006). Regulation of hypocretin (orexin) expression in embryonic zebrafish. J. Biol. Chem. 281, 29753–29761 10.1074/jbc.M605811200 16867991

[B21] Fernandes A. M. Fero K. Arrenberg A. B. Bergeron S. A. Driever W. Burgess H. A. (2012). Deep brain photoreceptors control light-seeking behavior in zebrafish larvae. Curr. Biol. 22, 2042–2047 10.1016/j.cub.2012.08.016 23000151 PMC3494761

[B22] Fernandez M. P. Berni J. Ceriani M. F. (2008). Circadian remodeling of neuronal circuits involved in rhythmic behavior. PLoS Biol. 6:e69 10.1371/journal.pbio.0060069 18366255 PMC2270325

[B23] Frenkel L. Ceriani M. F. (2011). Circadian plasticity: from structure to behavior. Int. Rev. Neurobiol. 99, 107–138 10.1016/B978-0-12-387003-2.00005-7 21906538

[B24] Gilestro G. F. Tononi G. Cirelli C. (2009). Widespread changes in synaptic markers as a function of sleep and wakefulness in Drosophila. Science 324, 109–112 10.1126/science.1166673 19342593 PMC2715914

[B25] Goldshmid R. Holzman R. Weihs D. Genin A. (2004). Aeration of corals by sleep-swimming fish. Limnol. Oceanogr. 49, 1832–1839

[B26] Gothilf Y. Coon S. L. Toyama R. Chitnis A. Namboodiri M. A. Klein D. C. (1999). Zebrafish serotonin N-acetyltransferase-2: marker for development of pineal photoreceptors and circadian clock function. Endocrinology 140, 4895–4903 10499549 10.1210/endo.140.10.6975

[B27] Granados-Fuentes D. Herzog E. D. (2012). The clock shop: coupled circadian oscillators. Exp. Neurol. [Epub ahead of print]. 10.1016/j.expneurol.2012.10.011 23099412 PMC3568450

[B28] Hartse K. M. (2011). The phylogeny of sleep. Handb. Clin. Neurol. 98, 97–109 10.1016/B978-0-444-52006-7.00007-1 21056182

[B29] Hendricks J. C. Finn S. M. Panckeri K. A. Chavkin J. Williams J. A. Sehgal A. (2000). Rest in Drosophila is a sleep-like state. Neuron 25, 129–138 10.1016/S0896-6273(00)80877-6 10707978

[B30] Hurd M. W. Cahill G. M. (2002). Entraining signals initiate behavioral circadian rhythmicity in larval zebrafish. J. Biol. Rhythms 17, 307–314 10.1177/074873002129002618 12164247

[B31] Hurd M. W. Debruyne J. Straume M. Cahill G. M. (1998). Circadian rhythms of locomotor activity in zebrafish. Physiol. Behav. 65, 465–472 9877412 10.1016/s0031-9384(98)00183-8

[B32] Karlsson K. A. Blumberg M. S. (2005). Active medullary control of atonia in week-old rats. Neuroscience 130, 275–283 10.1016/j.neuroscience.2004.09.002 15561443 PMC2630882

[B33] Karlsson K. A. Gall A. J. Mohns E. J. Seelke A. M. Blumberg M. S. (2005). The neural substrates of infant sleep in rats. PLoS Biol. 3:e143 10.1371/journal.pbio.0030143 15826218 PMC1079781

[B34] Kaslin J. Nystedt J. M. Ostergard M. Peitsaro N. Panula P. (2004). The orexin/hypocretin system in zebrafish is connected to the aminergic and cholinergic systems. J. Neurosci. 24, 2678–2689 10.1523/JNEUROSCI.4908-03.2004 15028760 PMC6729510

[B35] Kazimi N. Cahill G. M. (1999). Development of a circadian melatonin rhythm in embryonic zebrafish. Brain Res. Dev. Brain Res. 117, 47–52 10.1016/S0165-3806(99)00096-6 10536231

[B36] Lin L. Faraco J. Li R. Kadotani H. Rogers W. Lin X. (1999). The sleep disorder canine narcolepsy is caused by a mutation in the hypocretin (orexin) receptor 2 gene. Cell 98, 365–376 10.1016/S0092-8674(00)81965-0 10458611

[B37] Mehnert K. I. Beramendi A. Elghazali F. Negro P. Kyriacou C. P. Cantera R. (2007). Circadian changes in Drosophila motor terminals. Dev. Neurobiol. 67, 415–421 10.1002/dneu.20332 17443798

[B38] Meyer M. P. Smith S. J. (2006). Evidence from *in vivo* imaging that synaptogenesis guides the growth and branching of axonal arbors by two distinct mechanisms. J. Neurosci. 26, 3604–3614 10.1523/JNEUROSCI.0223-06.2006 16571769 PMC6673851

[B39] Mignot E. (2008). Why we sleep: the temporal organization of recovery. PLoS Biol. 6:e106 10.1371/journal.pbio.0060106 18447584 PMC2689703

[B40] Morris C. J. Aeschbach D. Scheer F. A. (2012). Circadian system, sleep and endocrinology. Mol. Cell. Endocrinol. 349, 91–104 10.1016/j.mce.2011.09.003 21939733 PMC3242827

[B41] Naumann E. A. Kampff A. R. Prober D. A. Schier A. F. Engert F. (2010). Monitoring neural activity with bioluminescence during natural behavior. Nat. Neurosci. 13, 513–520 10.1038/nn.2518 20305645 PMC2846983

[B42] Niell C. M. Meyer M. P. Smith S. J. (2004). *In vivo* imaging of synapse formation on a growing dendritic arbor. Nat. Neurosci. 7, 254–260 10.1038/nn1191 14758365

[B43] Nishino S. Sakurai T. (2006). The Orexin/Hypocretin System: Physiology and Pathophysiology. Towota, NJ: Humana Press

[B44] Noche R. R. Lu P. N. Goldstein-Kral L. Glasgow E. Liang J. O. (2011). Circadian rhythms in the pineal organ persist in zebrafish larvae that lack ventral brain. BMC Neurosci. 12:7 10.1186/1471-2202-12-7 21232144 PMC3031267

[B45] Pando M. P. Pinchak A. B. Cermakian N. Sassone-Corsi P. (2001). A cell-based system that recapitulates the dynamic light-dependent regulation of the vertebrate clock. Proc. Natl. Acad. Sci. U.S.A. 98, 10178–10183 10.1073/pnas.181228598 11517315 PMC56935

[B46] Panula P. (2010). Hypocretin/orexin in fish physiology with emphasis on zebrafish. Acta Physiol. (Oxf.) 198, 381–386 10.1111/j.1748-1716.2009.02038.x 19723028

[B47] Panula P. Chen Y. C. Priyadarshini M. Kudo H. Semenova S. Sundvik M. (2010). The comparative neuroanatomy and neurochemistry of zebrafish CNS systems of relevance to human neuropsychiatric diseases. Neurobiol. Dis. 40, 46–57 10.1016/j.nbd.2010.05.010 20472064

[B48] Piccinetti C. C. Migliarini B. Olivotto I. Coletti G. Amici A. Carnevali O. (2010). Appetite regulation: the central role of melatonin in Danio rerio. Horm. Behav. 58, 780–785 10.1016/j.yhbeh.2010.07.013 20692259

[B49] Prober D. A. Rihel J. Onah A. A. Sung R. J. Schier A. F. (2006). Hypocretin/orexin overexpression induces an insomnia-like phenotype in zebrafish. J. Neurosci. 26, 13400–13410 10.1523/JNEUROSCI.4332-06.2006 17182791 PMC6675014

[B50] Pyza E. Gorska-Andrzejak J. (2008). External and internal inputs affecting plasticity of dendrites and axons of the fly's neurons. Acta Neurobiol. Exp. (Wars) 68, 322–333 18511964 10.55782/ane-2008-1698

[B51] Raizen D. M. Zimmerman J. E. Maycock M. H. Ta U. D. You Y. J. Sundaram M. V. (2008). Lethargus is a Caenorhabditis elegans sleep-like state. Nature 451, 569–572 10.1038/nature06535 18185515

[B52] Rawashdeh O. De Borsetti N. H. Roman G. Cahill G. M. (2007). Melatonin suppresses nighttime memory formation in zebrafish. Science 318, 1144–1146 10.1126/science.1148564 18006748

[B53] Reppert S. M. Perlow M. J. Ungerleider L. G. Mishkin M. Tamarkin L. Orloff D. G. (1981). Effects of damage to the suprachiasmatic area of the anterior hypothalamus on the daily melatonin and cortisol rhythms in the rhesus monkey. J. Neurosci. 1, 1414–1425 7320754 10.1523/JNEUROSCI.01-12-01414.1981 PMC6564130

[B54] Rihel J. Prober D. A. Arvanites A. Lam K. Zimmerman S. Jang S. (2010). Zebrafish behavioral profiling links drugs to biological targets and rest/wake regulation. Science 327, 348–351 10.1126/science.1183090 20075256 PMC2830481

[B55] Saper C. B. Scammell T. E. Lu J. (2005). Hypothalamic regulation of sleep and circadian rhythms. Nature 437, 1257–1263 10.1038/nature04284 16251950

[B56] Scott E. K. Mason L. Arrenberg A. B. Ziv L. Gosse N. J. Xiao T. (2007). Targeting neural circuitry in zebrafish using GAL4 enhancer trapping. Nat. Methods 4, 323–326 10.1038/nmeth1033 17369834

[B57] Sehgal A. Mignot E. (2011). Genetics of sleep and sleep disorders. Cell 146, 194–207 10.1016/j.cell.2011.07.004 21784243 PMC3153991

[B58] Shapiro C. M. Hepburn H. R. (1976). Sleep in a schooling fish, Tilapia mossambica. Physiol. Behav. 16, 613–615 972954 10.1016/0031-9384(76)90222-5

[B59] Siegel J. M. (2005). Clues to the functions of mammalian sleep. Nature 437, 1264–1271 10.1038/nature04285 16251951 PMC8760626

[B60] Sigurgeirsson B. Thornorsteinsson H. Arnardottir H. Johannesdottir I. T. Karlsson K. A. (2011). Effects of modafinil on sleep-wake cycles in larval zebrafish. Zebrafish 8, 133–140 10.1089/zeb.2011.0708 21882999

[B61] Tamai T. K. Carr A. J. Whitmore D. (2005). Zebrafish circadian clocks: cells that see light. Biochem. Soc. Trans. 33, 962–966 10.1042/BST20050962 16246021

[B62] Tamai T. K. Young L. C. Whitmore D. (2007). Light signaling to the zebrafish circadian clock by Cryptochrome 1a. Proc. Natl. Acad. Sci. U.S.A. 104, 14712–14717 10.1073/pnas.0704588104 17785416 PMC1976231

[B63] Tauber E. Weitzman E. Korey S. (1969). Eye movements during behavioral inactivity in certain Bermuda reef fish. Commun. Behav. Biol. 3, 131–135

[B65] Tobler I. Borbely A. A. (1985). Effect of rest deprivation on motor activity of fish. J. Comp. Physiol. A 157, 817–822 3837116 10.1007/BF01350078

[B66] Tononi G. Cirelli C. (2006). Sleep function and synaptic homeostasis. Sleep Med. Rev. 10, 49–62 10.1016/j.smrv.2005.05.002 16376591

[B67] Tovin A. Alon S. Ben-Moshe Z. Mracek P. Vatine G. Foulkes N. S. (2012). Systematic identification of rhythmic genes reveals *camk1gb* as a new element in the circadian clockwork. PLoS Genet. 8:e1003116 10.1371/journal.pgen.1003116 23284293 PMC3527293

[B68] Vallone D. Gondi S. B. Whitmore D. Foulkes N. S. (2004). E-box function in a period gene repressed by light. Proc. Natl. Acad. Sci. U.S.A. 101, 4106–4111 10.1073/pnas.0305436101 15024110 PMC384702

[B69] Vallone D. Lahiri K. Dickmeis T. Foulkes N. S. (2005). Zebrafish cell clocks feel the heat and see the light! Zebrafish 2, 171–187 10.1089/zeb.2005.2.171 18248192

[B70] Vatine G. Vallone D. Appelbaum L. Mracek P. Ben-Moshe Z. Lahiri K. (2009). Light directs zebrafish period2 expression via conserved D and E boxes. PLoS Biol. 7:e1000223 10.1371/journal.pbio.1000223 19859524 PMC2759001

[B71] Vatine G. Vallone D. Gothilf Y. Foulkes N. S. (2011). It's time to swim! Zebrafish and the circadian clock. FEBS Lett. 585, 1485–1494 10.1016/j.febslet.2011.04.007 21486566

[B72] Vatine G. D. Zada D. Lerer-Goldshtein T. Tovin A. Malkinson G. Yaniv K. (2013). Zebrafish as a model for monocarboxyl transporter 8-deficiency. J. Biol. Chem. 288, 169–180 10.1074/jbc.M112.413831 23161551 PMC3537011

[B73] Vuilleumier R. Besseau L. Boeuf G. Piparelli A. Gothilf Y. Gehring W. G. (2006). Starting the zebrafish pineal circadian clock with a single photic transition. Endocrinology 147, 2273–2279 10.1210/en.2005-1565 16497800

[B74] Wang G. Grone B. Colas D. Appelbaum L. Mourrain P. (2011). Synaptic plasticity in sleep: learning, homeostasis and disease. Trends Neurosci. 34, 452–463 10.1016/j.tins.2011.07.005 21840068 PMC3385863

[B75] Whitmore D. Foulkes N. S. Sassone-Corsi P. (2000). Light acts directly on organs and cells in culture to set the vertebrate circadian clock. Nature 404, 87–91 10.1038/35003589 10716448

[B76] Yokogawa T. Marin W. Faraco J. Pezeron G. Appelbaum L. Zhang J. (2007). Characterization of sleep in zebrafish and insomnia in hypocretin receptor mutants. PLoS Biol. 5:e277 10.1371/journal.pbio.0050277 17941721 PMC2020497

[B77] Zhdanova I. V. (2005). Melatonin as a hypnotic: pro. Sleep Med. Rev. 9, 51–65 10.1016/j.smrv.2004.04.003 15649738

[B78] Zhdanova I. V. (2011). Sleep and its regulation in zebrafish. Rev. Neurosci. 22, 27–36 10.1515/RNS.2011.005 21615259

[B79] Zhdanova I. V. Wang S. Y. Leclair O. U. Danilova N. P. (2001). Melatonin promotes sleep-like state in zebrafish. Brain Res. 903, 263–268 10.1016/S0006-8993(01)02444-1 11382414

[B80] Zimmerman J. E. Naidoo N. Raizen D. M. Pack A. I. (2008). Conservation of sleep: insights from non-mammalian model systems. Trends Neurosci. 31, 371–376 10.1016/j.tins.2008.05.001 18538867 PMC2930986

[B81] Ziv L. Levkovitz S. Toyama R. Falcon J. Gothilf Y. (2005). Functional development of the zebrafish pineal gland: light-induced expression of period2 is required for onset of the circadian clock. J. Neuroendocrinol. 17, 314–320 10.1111/j.1365-2826.2005.01315.x 15869567

